# Cortical Anaplastic Ependymoma with Significant Desmoplasia: A Case Report and Literature Review

**DOI:** 10.1155/2013/354873

**Published:** 2013-12-16

**Authors:** Alaa Eldin Elsharkawy, Raid Abuamona, Markus Bergmann, Shadi Salem, Evariste Gafumbegete, Ernst Röttger

**Affiliations:** ^1^Neurosurgical Department, Ludmillenstift Hospital, Ludmillenstraße 4-6, 49716 Meppen, Germany; ^2^Neurosurgical Department, University of Kiel, 24105 Kiel, Germany; ^3^Department of Clinical Neuropathology, Bremen, Germany; ^4^Pathological Department, Ludmillenstift Hospital, Meppen, Germany

## Abstract

Ectopic brain anaplastic ependymomas with no connection to the ventricles are rare. We present a rare case of a 25-year-old male who presented with generalized convulsions. Computed tomography (CT), Magnetic Resonance Imaging (MRI), and magnetic resonance spectroscopy (MRS) showed characters of an intra- and extra-axial lesion. Intraoperatively, the lesion was a cortical solid mass that had no connections to the dura or to the ventricle. The histological diagnosis showed an anaplastic ependymoma with WHO grade III with distinctive desmoplasia. A literature review of ectopic anaplastic ependymomas regarding their clinical presentations, management, and prognostic factors was performed. There is a need to establish a clinically based histopathological grading system for anaplastic ependymomas. Ectopic anaplastic ependymomas should be included in the preoperative differential diagnosis.

## 1. Introduction

Ependymomas are a subtype of glioma that arise from the ependymal cells within the ventricles and the central canal of the spinal cord. These types of tumors account for 1.9% of all primary CNS tumors [1, 2].

Ependymomas arising outside of the ventricles and that do not have any connections to the ventricles specially cortical ependymomas are very rare [3].

Anaplastic ependymomas account for 8.6% to 11.5% of all ependymomas [1, 4]. The optimal histological grading scale for this type of tumor is not yet defined [5]. The postoperative management and prognostic factors are unknown.

We present a rare case of a cortical anaplastic ependymoma that preoperatively was not typical for intra-axial or extra-axial lesion. We reviewed all cases of ectopic anaplastic ependymomas without connections to the ventricles in the literature to gather information regarding their sites, clinical presentations, pathological features, management, and outcomes.

## 2. Case Report

A right-handed 25-year-old male presented with a generalized convulsion. He reported having focal-like seizures for years without treatment. He reported slight numbness in his left fourth and fifth fingers. No other symptoms or neurological deficits were present.

**Figure 1:**
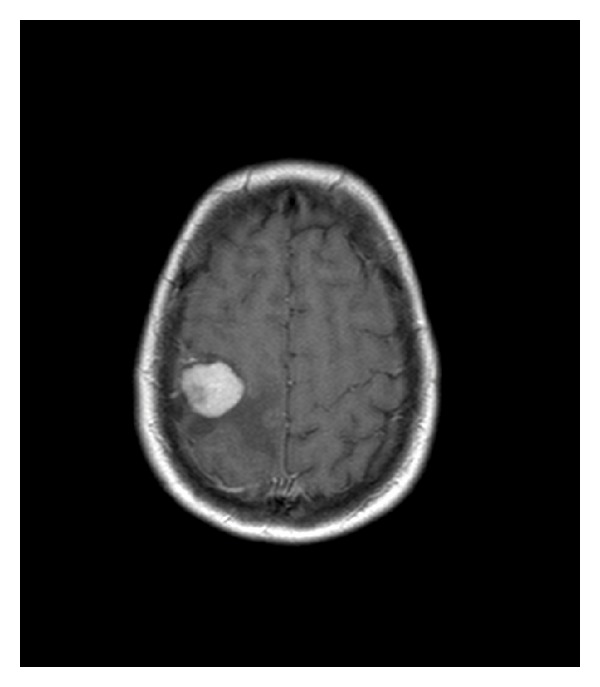
Preoperative MRI showing the mass in the right frontal lobe.

The initial brain CT revealed a mass in the right central area that appeared to be a meningioma. An MRI 3-Tesla scan of the brain demonstrated a solid, well-demarcated homogeneous mass which may have a dural attachment (Figure 1). An MRS scan showed a 125% increase in choline. N-Acetyl-aspartate and keratin/phospho-keratin were not observed to be present in the tumor. There was no increased resonance in the lipidarea, and there was no alanine peak. The choline monopeak in the tumor corresponds to an extra-axial tumor, and the lack of resonance in the lipid area confirms the diagnosis of an extra-axial tumor and also excludes a metastasis (Figure 1).

### 2.1. Intervention

A frontoparietal craniotomy was performed with MRI-guided navigational assistance. Grossly, the lesion was a cortical firm, gray, and solid mass with no connection to the dura matter. The gross lesion appearance and separation from the dural matter allowed for clear planes of dissection between the tumor and normal brain tissue. Intraoperatively, the tumor was not found to be connected to the ventricle. A gross total resection of the mass was achieved. Postoperatively, the patient had no deficits. The preoperative light numbness in his left hand improved during the hospital stay. After diagnosis of an anaplastic ependymoma, an MRI of the total neural axis was performed, without evidence of droop metastasis.

### 2.2. Pathological Examination

On histological examination, the tumor was primarily of high cellularity and had sharp borders with the surrounding CNS tissue, in which piloid gliosis with rosenthal fibers was seen. The tumor cells were diffusely distributed with little fibrillary intercellular substance. The tumor cell nuclei had finely dispersed chromatin with moderate anisonucleosis and some giant nuclei. Mitotic activity was increased, with up to four mitotic figures seen per high-power field, 11/10 hpf. In fields of lower cellular density, perivascular pseudorosettes were seen. Gemistocytic cells and calcifications were distributed throughout the tumor tissue. Focally, the tumor tissue contained many collagenous fibers.

Immunohistochemically, the tumor cells expressed GFAP, S100, and vimentin. An anti-EMA reaction showed dot-like staining of the microlumina. CD34 was only expressed in the endothelial cells. Thirty percent (30%) of the tumor cell nuclei showed a positive anti-Ki67 reaction.

**Figure 2:**
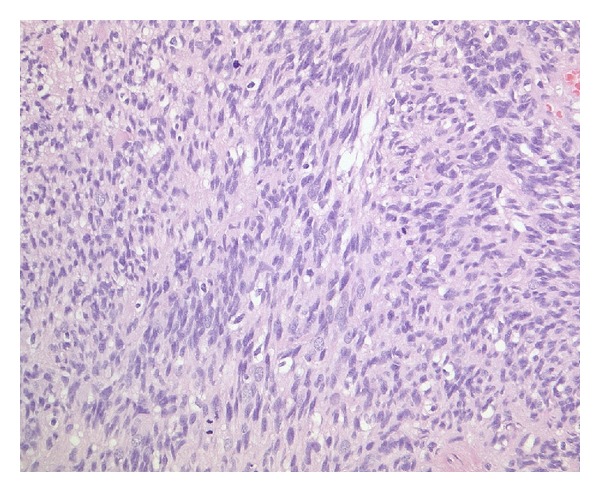
A patternless, highly cellular lesion is seen with increased mitotic activity (H&E, ×200, original magnification).

**Figure 3:**
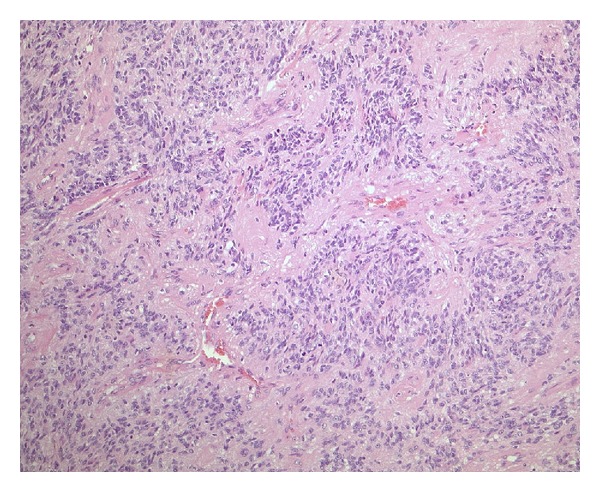
Sheets of monomorphic cells were interrupted by perivascular pseudorosettes (H&E, ×200, original magnification).

**Figure 4:**
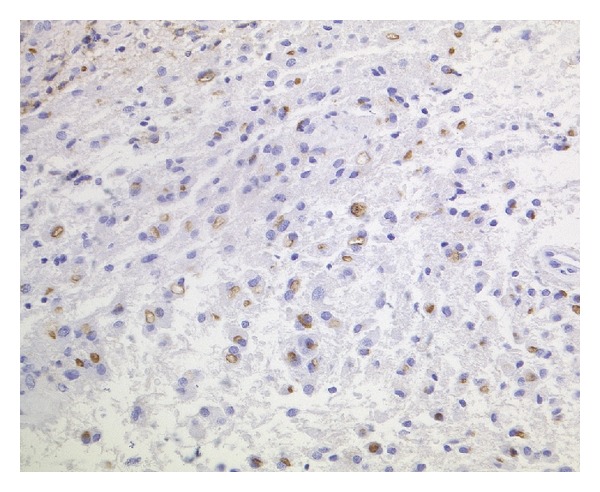
Paranuclear and ring-like structures were seen in the anti-EMA reaction (anti-EMA, ABC, ×200, original magnification).

**Figure 5:**
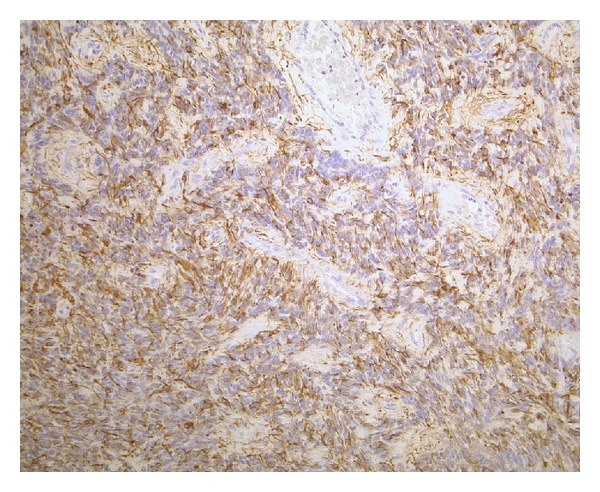
Tumor cell processes were GFAP-positive and built up perivascular pseudorosettes (anti-GFAP, ABC, ×200, original magnification).

**Figure 6:**
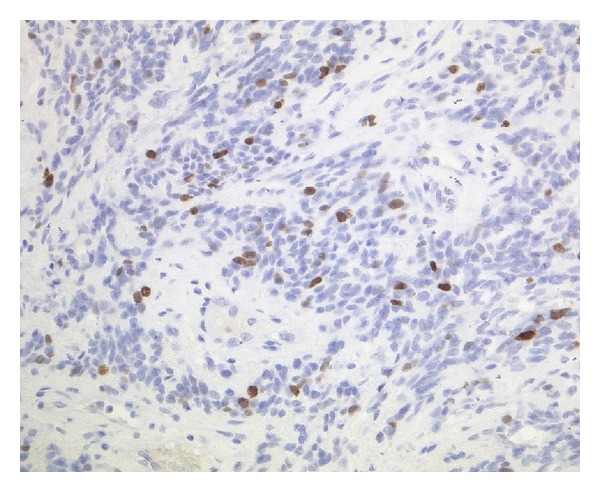
Ki67-index was increased, with up to 30% positive nuclei (anti-Ki67, ABC, ×200, original magnification).

On electron microscopy, some microlumina and remnants of cilia were detected (Figures 2, 3, 4, 5, and 6).

The final diagnosis was an anaplastic ependymoma with WHO grade III and distinctive desmoplasia (Figures 2, 3, 4, 5, and 6).

### 2.3. Postoperative Follow-Up

**Figure 7:**
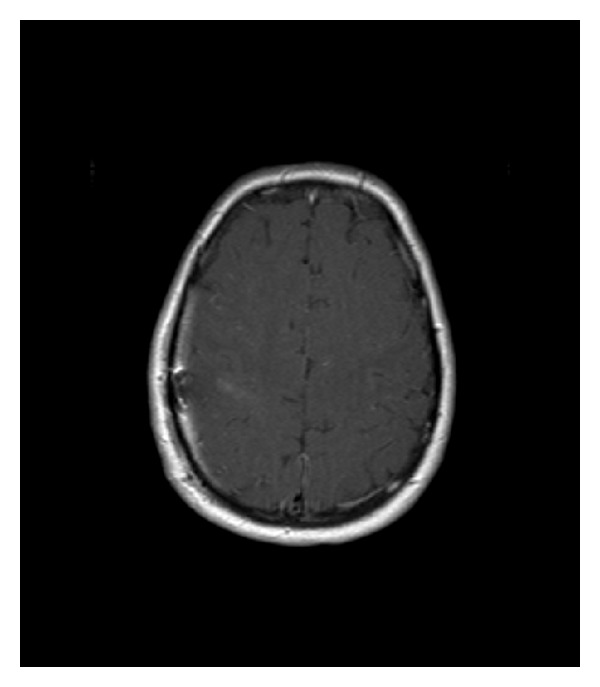
Postoperative MRI showing that the tumor has been totally removed.

Follow-up at 6 months revealed that the patient had well-controlled epileptic seizure activity, and an MRI of the brain showed no evidence of residual tumor or tumor recurrence. Adjuvant local radiotherapy was performed (Figure 7).

### 2.4. A Literature Review of Ectopic Anaplastic Ependymomas with No Connection to the Ventricle

We identified 24 cases of ectopic anaplastic ependymomas with no connections to the ventricle. There were 13 males (54.2%) and 11 females (45.8%), and the ages ranged between 0.3 and 70 years (mean 28.8 years). Of the 24 ectopic anaplastic ependymoma cases, 22 were located supratentorially (91.7%) and 2 were located infratentorially (8.3%). Nineteen cases (79.2%) were intra-axial and 5 (20.8%) were extra-axial ependymomas. The location, clinical presentation, radiological finding, and outcomes are summarized in Table 1.

The frontal location was dominant, being reported in 11 cases (45.9%), including 2 frontoparietal, one frontotemporal, and one central. The most often reported clinical presentation was seizure and medically intractable epilepsy. The most often reported radiological appearance was solid with cystic formation. Preoperatively, 2 cases mimicked a meningioma, and one case mimicked a glioblastoma. Of the 24 anaplastic ependymomas, there was one case in which the exact grading is debatable [6]. Surgical excision was the standard in all of the cases, except in the case involving the brainstem, in which only a biopsy was performed. The majority of the cases were treated with radiotherapy following the surgery.

Of the 24 cases, 12 (50%) patients had no postoperative deficits, 2 (4.2%) had mild deficits, one (4.2%) patient was handicapped, and one (4.2%) patient was bedridden. Six patients (27.3%) died during the follow-up period.

## 3. Discussion

Ectopic brain ependymomas that do not have any connection to the ventricular system have been reported in all regions of the brain [7–13].

Our unique case had isointense signals on T1- and T2-weighted images and homogeneous contrast enhancement with no dural tail sign. The MRS showed characteristics of an extra-axial lesion. Our case reflects the difficulty in differentiating ectopic anaplastic ependymomas from other dural-based extra-axial lesions and other gliomas on the basis of signal characteristics alone. Due to the rarity of ectopic ependymomas, these tumors are generally not included in the differential diagnosis. Several authors reported anaplastic ependymomas that mimicked meningiomas [7, 14].

Despite the malignant designation of anaplastic ependymomas, they tend to be solid and well demarcated with limited infiltration to the edges of the lesion. The diagnosis of an ectopic anaplastic ependymoma is not easy. The diagnosis varies widely depending on the pathologist's experience with ependymomas [15–17]. It was reported that approximately 15% of anaplastic ependymoma had a prior diagnosis other than ependymoma, and the tumors were subsequently reclassified as ependymoma [18]. The lack of clinicohistopathological concordance highlights the need for establishing criteria for classifying these tumors according to their degree of anaplasticity.

Surgical excision with adjuvant radiotherapy is the primary management of anaplastic ependymomas [10, 19, 20]. Surgical treatment alone [8, 21, 22] and postoperative combined chemotherapy and radiotherapy were also reported [6, 12, 22, 23]. Chemotherapy has shown only limited efficacy [11]. Chemotherapy may be indicated in cases of incomplete surgical resection and in the pediatric group under the age of two [24, 25].

The 5- and 10-year survival rates reported in the literature were 65% und 37%, respectively, with a great disparity among the studies [22, 29–31]. Certain authors have suggested that supratentorial tumors are more biologically aggressive or recur earlier than infratentorial lesions. Long-term survival appears to be similar for the two locations [22].

Site-related outcomes were reported with the worst outcomes for intraparenchymal anaplastic ependymomas [30]. Successful gross total resection appears to be the best prognostic indicator of long-term survival [15, 31–34]. Favorable prognostic factors reported were older age, a higher local radiation dose, and Caucasian race [35]. Decreased overall survival was reported in cases in which the patient's age was younger than 15 years, subtotal resection was performed, and adjuvant therapy was used [5].

An increased risk of recurrence was reported with a high histological grade, incomplete resection, and a Karnofsky performance status that is less than or equal to 80 [36].

Tumor grade as a prognostic factor was contradictory; certain authors reported that tumor grade was an independent prognostic factor that influences outcome [37], and others reported that outcomes were not affected by histological grade [22].

## 4. Conclusion

There is a need to establish a clinically based histopathological grading system for anaplastic ependymomas and a need to increase the awareness of these lesions during preoperative studies.

## Figures and Tables

**Table 1:** All of the ectopic anaplastic ependymomas with no connection to the ventricles reported before January 2012.

Series	Sex/age	Location	Clinical presentation	Surgery	Postop. treatment	Recurrence	Outcome	Follow-up
Alexiou et al. [21]	F/10	Rt. Frontal	Headache	GTE	NAT	Cortical	No deficits	6
Davis et al. [19]	F/22	Rt. Frontotemporal	Headache	GTE	Radiotherapy	Metastasis to scalp	No deficits	60
Fukui et al. [13]	M/66	Lt. Posterior fossa	Nerve deficit	GTE	Radiotherapy	Extra-axial cerebellar	No deficits	30
Hamano et al. [11]	M/15	Lt. Parieto-occipital	Headache	GTE	Chemotherapy	Parenchymal with cortical extension	No deficits	12
Kojima et al. [26]	F/56	Lt. Temporal	Seizure	STE	Radiotherapy	Pure cortical	No deficits	5
Kutlay et al. [10]	F/11	Lt. Frontoparietal	Seizure	STE	Radiotherapy	cortical	—	
Miyazawa et al. [23]	M/33	Lt. Parietal	Headache	STE	Combined	Extra-axial intratumoral hemorrhage	Mild deficits	10
Moritani et al. [6]	F/50	Rt. Temporal	Headache	STE	Combined	Recurrence in 20 m the exact grading is debatable	—	
Ng et al. [27]	F/51	Bifrontal	Incidental	GTE	Radiotherapy	Parenchymal with cortical extension	Handicapped	8
Niazi et al. [15]	F/36	Rt. Frontal	Seizure	GTE	Radiotherapy	Parenchymal with cortical extension	No deficits	29
Niazi et al. [15]	F/18	Rt. Frontoparietal	Seizure	GTE	Radiotherapy	Recurrence	Death	14
Ohla et al. [28]	M/29	Lt. Parietal	Seizure	STE	Radiotherapy	Parenchymal with cortical extension	Death	
Park et al. [7]	F/17	Rt. Parafalcine, falx	Seizure	GTE	Radiotherapy	Extra-axial meningioma	Mild deficits	2
Romero et al. [9]	M/23	Lt. Frontal	Seizure	GTE	Radiotherapy	Pure cortical	No deficits	60
Singh et al. [14]	M/35	Lt. Parafalcine, falx	Seizure	GTE	Radiotherapy	Extra-axial meningioma	No deficits	12
Takeshima et al. [8]	F/70	Rt. Frontal	Loss of consciousness	GTE	NAT	Extra-axial intratumoral hemorrhage	Bedridden	36
Thakar et al. [12]	M/12	Brainstem	Headache	Biopsy	Combined	Brainstem	Death	1
Van Gompel et al. [20]	M/12	Rt. Parietal	Seizure	GTE	Radiotherapy	Unusual epileptogenic	No deficits	101
Van Gompel et al. [20]	M/25	Rt. Frontal	Seizure	GTE	Radiotherapy	Unusual epileptogenic	No deficits	80
Van Gompel et al. [20]	M/59	Rt. Frontal	Seizure	GTE	Radiotherapy	Unusual epileptogenic	No deficits	47
Vinchon et al. [22]	M/15	Lt._Insular	Headache	STE	Combined	Recurrence in 3 m	Death	14
Vinchon et al. [22]	M/0.3	Rt. Central	Headache	STE	NAT	Recurrence in 5 m	Death	6
Vinchon et al. [22]	F/13.5	Rt. Temporal	Headache	GTE	Radiotherapy	Recurrence in 8 m	Death	11
Vinchon et al. [22]	M/11.3	Rt. Parietal	Seizure	GTE	Radiotherapy	Recurrence in 20 m	No deficits	80
Present-case	M/25	Rt. Frontal	Seizure	GTE	Radiotherapy	Extra-axial Meningioma	No deficits	6

Lt.: left, Rt.: right, M: male, F: female, GTE: gross total excision, STE: Subtotal excision, NAT: no adjuvant therapy, ND: no deficits.
